# Effect of Zedo Gum‐Based Coatings Containing Tarragon and *Zataria multiflora* Boiss Essential Oils on Oil Uptake, Acrylamide Formation and Physicochemical Properties of Fried Potato Strips

**DOI:** 10.1002/fsn3.70347

**Published:** 2025-05-23

**Authors:** Niyoosha Khajeh, Hamid Babapour, Bahram Hassani, Abdorreza Mohammadi Nafchi, Leila Nouri, Ahmadreza Abedinia

**Affiliations:** ^1^ Food Science and Technology Department, Da.C. Islamic Azad University Damghan Iran; ^2^ Department of Agricultural Engineering National University of Skills (NUS) Tehran Iran; ^3^ Department of Food Industry, Faculty of Agriculture Ferdowsi University of Mashhad Mashhad Iran

**Keywords:** acrylamide, edible coating, oil uptake, oxidative stability, potato strips, tarragon, *Zataria multiflora* Boiss

## Abstract

Fried foods are popular globally, but their high oil absorption, lipid oxidation, and toxic substances, particularly acrylamide, during deep‐fat frying pose health risks to consumers. This study aimed to investigate the possibility of using an active coating based on zedo gum containing *Zataria multiflora* Boiss (ZE) and tarragon essential oils (TE) to maintain the quality of fried potatoes. Zedo gum coating (1% v/w) containing 1% (v/v) essential oils was prepared and potato strips were immersed in the produced coating solutions for 5 min. The strips were deep‐fried in a fryer at 180°C for 5 min, and then physicochemical, oxidative and sensory tests were performed on the samples. The coated strips experienced significantly lower oil uptake, moisture loss, acrylamide formation, and lipid oxidation during frying compared to the control. Additionally, coatings improved the total phenol content, antioxidant activity, and color of the strips (*p* < 0.05). The quality, acceptance, and shelf life of fresh and fried potato strips were enhanced. The highest TPC (40.23 mg GAE/g), antioxidant activity (45.94%), moisture content (64.16%) and the lowest amount of acrylamide (89.84 μg/kg) were observed in ZE coated strips, and this treatment also had low oil content (9.23%) and the highest oxidative stability (in terms of peroxide, anisidine, TBA, and acid values) and scored high sensory acceptance; it was selected as the best treatment in this study.

## Graphical abstract

Zedo gum coatings with *Zataria multiflora* Boiss and tarragon essential oils reduce oil uptake, acrylamide formation, and lipid oxidation in fried potatoes. Coated potato strips exhibit enhanced antioxidant activity, total phenol content, and improved sensory attributes. The active coating improves the quality, safety, and shelf life of fresh and fried potato strips, offering a healthier fried food option.

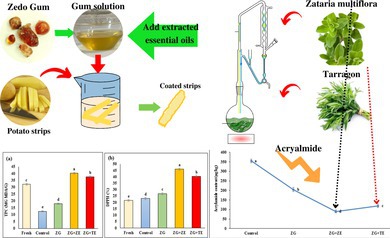

## 1 Introduction

Fried potatoes, widely consumed as snack food products, have gained substantial popularity among children, teenagers, and adults alike due to their appealing taste and texture (Esmaeili et al. 2024). Deep frying is a widely used cooking method in the food industry, renowned for its crispness, golden color, and desirable flavor (Devi et al. 2021). Frying, despite its palatability benefits, can lead to oxidative degradation of lipids and harmful compounds, posing health risks such as obesity, diabetes, cardiovascular diseases, and metabolic disorders (Lumanlan et al. 2020). Additionally, oxidative degradation of frying oil leads to the generation of free radicals and toxic compounds, which further deteriorate food quality and pose health risks (Moufakkir et al. 2023).

Acrylamide, a harmful compound formed during frying, is primarily found in carbohydrate‐rich foods through the Maillard reaction, exhibiting carcinogenic and neurotoxic properties (Orsák et al. 2022; Wu et al. 2022). The presence of acrylamide in fried potato products raises food safety concerns due to potential cancer and neurological disorders linked to prolonged dietary exposure (Knight et al. 2021). Research on reducing oil uptake and acrylamide formation in fried foods is crucial for improving nutritional quality and safety. Biopolymer‐based edible coatings offer promising solutions (Wang, Ng, et al. 2023; Xie et al. 2022). Edible coatings are thin, biodegradable films made from polysaccharides, proteins, or lipids that can be applied directly to food surfaces to regulate moisture transfer, delay oxidation, and enhance quality (Yong and Liu 2021). Additionally, these coatings serve as effective carriers for bioactive compounds such as antioxidants and antimicrobial agents, further enhancing the stability and shelf life of food products (Babapour et al. 2022). Zedo gum exudates from *Amygdalus scoparia*, a natural arabinogalactomannan, offer a cost‐effective, non‐toxic, and functional gum with superior emulsifying and gelling capabilities, making it ideal for fried food coating applications (Razmjoo et al. 2021).

Gölükcü et al. (2024) and Gorzin et al.'s (2024) studies highlight the diverse chemical composition of essential oils (EOs) in the market. In 2022, EO imports reached 31,783,450 USD, while exports exceeded imports at 29,599,149 USD. EOs are known for their antioxidant and antimicrobial properties. The composition of EOs varies based on cultivar, harvesting time, and extraction method. The duration of hydrodistillation also influences the composition and yield of essential oils like bergamot peel essential oil (Bozova et al. 2024). Sharma et al.'s (2021) study of *Azadirachta indica* EOs from North India also reveals significant variations in their chemical profile.

Tarragon ( *Artemisia dracunculus* L.), an aromatic herb from the Asteraceae family, is used in culinary and traditional medicine due to its antioxidant and antimicrobial properties (Azizkhani et al. 2021; Ekiert et al. 2021). Similarly, *Zataria multiflora* Boiss (Shirazi thyme), a Lamiaceae plant, is known for its antimicrobial, antifungal, and antioxidant properties due to its rich composition of thymol and carvacrol (Hematizad et al. 2021; Karimi and Meiners 2021). These EOs have been widely explored and have shown promising effects in mitigating lipid oxidation and acrylamide formation in fried foods (Ahmadi‐Dastgerdi et al. 2022; Hashemi et al. 2021).

Given the increasing demand for healthier and safer fried food products, this study aims to investigate the potential of zedo gum‐based edible coatings containing tarragon and *Z. multiflora* essential oils in reducing oil uptake, lipid oxidation, acrylamide content, and physicochemical degradation in fried potato strips. By leveraging the combined effects of biopolymer‐based coatings and natural bioactive compounds, this research seeks to contribute valuable insights into sustainable and health‐conscious food processing techniques.

## 2 Materials and Methods

### 2.1 Materials

The zedo gum and potatoes (Agria variety) were purchased from the Giahine Co. of Esfahan (Iran) and the local market of Tehran (Iran), respectively. The potatoes (with an approximate weight of 150–200 g) used in this study were grown in the temperate climate of the Esfahan region, which experiences a semi‐arid climate with hot summers and cold winters. The annual average temperature in this region is approximately 16°C, and the average annual precipitation is around 200 mm. The chemicals used to perform tests were prepared from Merck Co. of Germany.

### 2.2 Preparation of *Z. multiflora* Boiss (ZE) and Tarragon Essential Oils (TE)

Tarragon and *Z. multiflora* Boiss were collected from Agricultural University lands (Damghan, Iran), and their leaves were separated. Both plants were cultivated under semi‐arid climatic conditions, characterized by an average annual temperature of 16°C–18°C, low precipitation (150–200 mm/year), and sandy loam soil with good drainage. The plants were grown without the use of chemical pesticides or fertilizers. Tarragon was harvested in mid‐spring (May), while *Z. multiflora* Boiss was collected in late summer (August) at the full flowering stage to ensure maximum essential oil yield and quality. The leaves were dried in a dark place at ambient temperature for 1 week. The Tarragon (TE) and *Z. multiflora* Boiss (ZE) essential oils were prepared using the Clevenger system and water distillation method. The ratio of distilled water to plant powder and the time of extraction were 7:1 and 3 h, respectively (Zaïri et al. 2020).

### 2.3 Preparation of Edible Coatings and Fried Potato Strips

To develop the novel zedo gum‐based edible coating, an optimized concentration of 1% (w/v) of gum was added to the deionized water and stirred for 30 min. The solution was then kept for a day at room temperature. The gum solution was centrifuged at 3300×*g* for 14 min (Sigma 1–14, Germany), and then the supernatant was collected and applied for coating. The selection of zedo gum concentration and processing conditions was based on preliminary trials (data not shown), ensuring the formation of a stable and effective coating. To enhance the functional properties of the coating, the bioactive components tarragon EO (TE) and *Z. multiflora* Boiss EO (ZE) were incorporated at 1% (v/v) concentrations, selected based on their antimicrobial and antioxidant effectiveness in previous studies. The emulsification process was conducted using a high‐speed homogenizer (IKA SS42, Germany) at 12,000 rpm for 5 min to ensure the uniform dispersion of essential oils in the gum matrix (Barzegar et al. 2019). This formulation aimed to develop an innovative coating system capable of reducing oil uptake, acrylamide formation, and lipid oxidation while improving the physicochemical properties of fried potato strips. For sample preparation, four sets of potato strips were considered:

1. Control (Uncoated)—Potato strips immersed in distilled water.
2. ZG (Zedo Gum Coating Only)—Potato strips coated with 1% (w/v) zedo gum solution.
3. ZG + ZE (Zedo Gum with *Z. multiflora* Essential Oil)—Potato strips coated with zedo gum solution containing 1% (v/v) ZE.
4. ZG + TE (Zedo Gum with Tarragon Essential Oil)—Potato strips coated with zedo gum solution containing 1% (v/v) TE.

Initially, the washed and peeled potatoes were cut into 1 × 1 × 8 cm strips with the cutter, and then in order to remove starch, the strips were washed and immersed in distilled water. The strips were then boiled for 6 min at 85°C to remove the starch. After that, the potato strips were immersed in zedo gum‐based edible coatings for 5 min in the ratio of 1 g of potato strips to 5 mL of edible coating solution. The strips immersed in distilled water were considered as control. The strips were then dried at ambient temperature. The strips were deep‐fried in frying oil and in a Tefal fryer (France) at 180°C for 5 min. The ratio of strips to frying oil was 1:6. After the frying process, the strips were cooled on an oil absorption pad (Yakend, YR20, Iran) and clean cloth at room temperature.

### 2.4 Moisture and Oil Content Determination

To determine the moisture amounts of fresh and fried potato strips, the pureed samples were dried at 105°C ± 1°C in a Memmert oven (Germany) until the constant weight was reached. The oil content of fried potatoes was determined using diethyl ether solvent and the continuous Soxhlet extraction. The temperature and time of extraction were 150°C and 4 h, respectively (AOAC 2005).

### 2.5 Total Phenol Content (TPC) and Antioxidant Activity

The TPC of the fried and fresh potatoes was measured using the standard method of Folin–Ciocalteu and gallic acid standard curve (*Y* = 0.1575 × −0.1429; *R* ^2^ = 0.9992) (Awad et al. 2021). The DPPH radical scavenging assay at 515 nm was used to determine the antioxidant activity of potato strips (Licciardello et al. 2018).

### 2.6 Oxidative Index Determination

To investigate the oxidative stability of fried potato strips, first the oil of each sample was extracted and kept in an oven at 20°C for 30 days, and every 15 days, their oxidation indexes, including peroxide value (PV), para anisidine value (p‐AnV), thiobarbituric acid index (TBA) and acid value (AV), were tested. The PV of oils was determined using the titration method with 0.01 N sodium thiosulphate (AOCS 2003), p‐AnV was measured using the spectroscopic method (UV–visible s2150, Unico, America) at 350 nm (Gebremeskel et al. 2022), TBA was assessed using the spectroscopic method at 530 nm, and AV was measured using the titration method with 0.1 N KOH (Javani‐Seraji et al. 2023).

### 2.7 Acrylamide Content Determination

High performance liquid chromatography (HPLC; Knauer, Germany) was used to extract acrylamide in fried potato strips. Initially, the samples (1 g) were extracted using 10 mM formic acid in three stages (20 mL) and after cold centrifuging for 10 min at 2370×*g*, the extracted colloids were settled using carrez solution, and the obtained extracts were purified using an Oasis MCX cartridge. To analyze the samples, an HPLC system with a TQ detector and UPLC HSS T3 column was used. Formic acid (10 mM) and methanol (0.5%) were used as the mobile phase. The mobile phase flow rate, nitrogen gas flow rate, and the temperature of the column and atomizer were set at 0.3 mL/min, 900 L/h, 40°C, and 10°C, respectively. The amount of acrylamide in the samples was determined in μg/kg using an acrylamide calibration curve (*Y* = 47,200x + 42,000, *R* ^2^ = 0.9967) (Gökmen et al. 2009). The concentration value was taken into consideration while estimating the limit of detection (LOD) and limit of quantification (LOQ), which produced signals that were 3 and 10 times the noise signal, respectively (Ghalebi et al. 2019).

### 2.8 Hardness Determination

The hardness of fresh and fried potato strips (highest breaking force) was analyzed with a texture analyzer (TA‐XT2i, Stable Micro Systems, Surrey, U.K.) at 25°C ± 1°C. In this test, the strips were compressed to 55% of their initial height using a cylinder probe (50 mm diameter) at a speed of 1 mm/s. The hardness was the maximum applied force, which was reported in newton (N) (Esmaeili et al. 2024).

### 2.9 Color Determination

The color of fresh and fried potato strips was analyzed using a Color Flex colorimeter (America) and the color indexes including L* (0 = black and 100 = white), a* (negative = green and positive = red), b* (negative = blue and positive = yellow) were determined, Chroma (C*), and Hue angle (*h*°) were computed using Equations (1) and (2), respectively (Kurek et al. 2021).

(1) $$C*=\sqrt{{a^{*}}^{2}+{b^{*}}^{2}}$$

(2) $$h^{{}^{\circ}}=\tan^{-1}\left(\frac{b^{*}}{a^{*}}\right)$$

### 2.10 Sensory Evaluation

Evaluation of the sensory characteristics of fried potato strips, including color, flavor, texture, and overall acceptability, was done using the five‐point Hedonic scale. First, the samples were randomly coded, and then they were given to the trained panelists (15 men and 15 women in the age range of 24–40 years) along with the questionnaire. The samples were coded, and between each sample evaluation, the panelists drank distilled water. The score of 1 indicates a very bad sample, and a score of 5 indicates a very good sample (Esmaeili et al. 2024).

### 2.11 Statistical Analysis

All tests were conducted in triplicates, and the obtained results were expressed as mean ± standard deviation (SD). The one‐way analysis of variance (ANOVA) was employed to determine the significant differences among the experimental groups. This statistical method was chosen because it is well‐suited to comparing the means of more than two groups (in our case, the control and different coating treatments). One‐way ANOVA helps in identifying whether there are statistically significant differences in the parameters under study, such as oil uptake, acrylamide formation, antioxidant activity, and other physicochemical properties of the fried potato strips. The significance level for all statistical tests was set at *p* < 0.05, and post hoc analysis was performed using Duncan's multiple range test. This post hoc test is commonly used following ANOVA to compare all pairs of means and to determine which specific group differences are statistically significant. The SPSS 22.0 software was used for all statistical analyses.

## 3 Results and Discussion

### 3.1 Effect of Zedo Gum‐Based Coatings on Moisture and Oil Content of Fried Potato Strips

**FIGURE 1:**
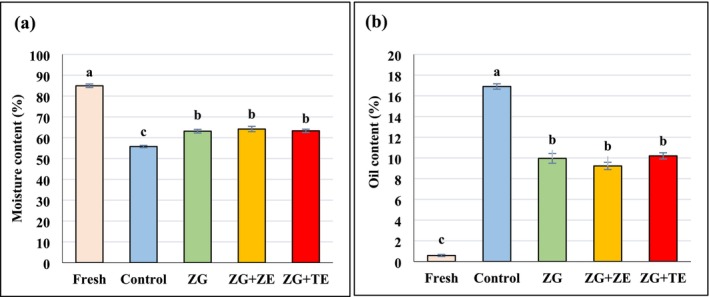
(a) Moisture content, (b) oil content of fresh and fried potato strips. Different letters represent significant differences at the 5% level of probability among samples. ZG: Zedo gum‐based coating; ZG + ZE: Zedo gum‐based coating containing *Zataria multiflora* essential oil; ZG + TE: Zedo gum‐based coating containing tarragon essential oil.

The frying process significantly influenced both the moisture and oil content of potato strips, as shown in Figure 1a–c (*p* < 0.05). Before frying, the potato strips exhibited the highest moisture content of 84.93% and the lowest oil content of 0.58%. However, during the frying process, a significant decrease in moisture content was observed, while the oil content increased (*p* < 0.05). This can be attributed to the fact that during frying, thermal energy is absorbed by the potato strips, causing moisture on the surface to evaporate. The formation of a hard surface layer on the product then restricts moisture migration, leading to a greater loss of surface moisture due to pressure buildup and the creation of voids (Mai Tran et al. 2007; Mao et al. 2024).

This pressure differential, created within the product, forces water to migrate from the interior to the surface, and the surface moisture is then absorbed during frying. Simultaneously, the oil penetrates the voids created by evaporating moisture, replacing the water in the potato tissue (Debnath et al. 2003; Manoharan et al. 2024). As a result, the uncoated control potato strips exhibited the lowest moisture content (55.76%) and the highest oil content (16.90%), confirming the significant impact of the frying process on these parameters.

#### 3.1.1 Impact of Zedo Gum Coating on Moisture Retention and Oil Absorption

The application of zedo gum‐based coatings, either with tarragon extract (TE) or *Z. multiflora* Boiss extract (ZE), led to a significant reduction in moisture loss and oil absorption after frying compared to the uncoated control (*p* < 0.05). Both coated groups demonstrated comparable moisture retention and oil absorption rates, indicating that the zedo gum coating itself, rather than the inclusion of the essential oils, played a dominant role in preserving moisture and reducing oil uptake. Hydrocolloid‐based coatings, such as zedo gum, form a gel‐like structure when hydrated, which helps to prevent moisture evaporation and restricts oil penetration. This results in better moisture retention and a reduction in oil absorption during the frying process. Specifically, the uniform coating formed by zedo gum prevents the formation of pores caused by water vapor exit and reduces the capillary action that typically facilitates oil uptake into the potato tissue during frying (Ansarifar et al. 2018; Yadegari et al. 2017). This mechanism is consistent with previous studies that have reported similar effects of hydrocolloid coatings in reducing oil absorption and improving moisture retention in fried foods (Kurek et al. 2021; Zamani‐Ghalehshahi and Farzaneh 2021). The zedo gum coating works by acting as a barrier that limits the oil's access to the food tissue, thus minimizing its absorption and resulting in a healthier product with better sensory properties.

#### 3.1.2 Effect of Essential Oils on Moisture and Oil Uptake

The incorporation of tarragon and *Z. multiflora* essential oils into the zedo gum coating did not significantly alter the moisture retention or oil absorption properties compared to the uncoated control or the zedo gum alone. While essential oils are known for their antimicrobial and antioxidant properties, the primary function of the zedo gum coating in this context appears to be the prevention of oil absorption and moisture loss, as supported by previous research on edible coatings (Zokaei et al. 2020). However, the presence of essential oils may provide additional benefits in terms of flavor, aroma, and potential health benefits, which could be explored further in future studies. In conclusion, zedo gum‐based coatings effectively reduced oil absorption and moisture loss in fried potato strips, with no significant difference observed between the coatings containing tarragon and *Z. multiflora* essential oils. These findings suggest that zedo gum's barrier properties are the key factor in improving the quality of fried potato products. The results align with previous studies that highlight the effectiveness of hydrocolloid coatings in maintaining the quality of fried foods and enhancing their nutritional profile (Daraei Garmakhany et al. 2014; Zamani‐Ghalehshahi and Farzaneh 2021).

### 3.2 TPC and Antioxidant Activity of Fried Potatoes

**FIGURE 2:**
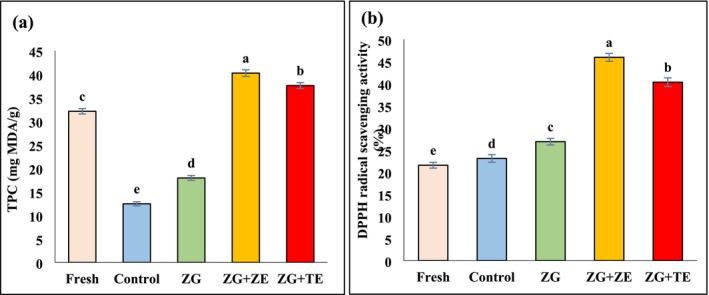
(a) TPC, (b) DPPH radical scavenging of fresh and fried potato strips. Different letters represent significant differences at the 5% level of probability among samples. ZG: Zedo gum‐based coating; ZG + ZE: Zedo gum‐based coating containing *Zataria multiflora* essential oil; ZG + TE: Zedo gum‐based coating containing tarragon essential oil. TPC, total phenol content.

Phenolic compounds, known for their antioxidative properties, play a pivotal role in preventing lipid oxidation and neutralizing free radicals. These bioactive molecules, present in ZE and TE essential oils, exhibit hydrogen‐donating capacity, which helps in stabilizing free radicals and delaying oxidation during frying (Šamec et al. 2014; Wang, Sun, et al. 2023). Our findings revealed that the application of zedo gum‐based coatings significantly increased the total phenolic content (TPC) and antioxidant activity of fried potato strips, thus indicating a promising protective mechanism. Although heat‐sensitive phenols may undergo degradation during the frying process, the zedo gum‐based coatings appeared to reduce this loss, likely by forming a protective barrier that limited oxidation and thermal breakdown. The control sample exhibited the lowest TPC (12.46 mg GAE/g), while the coated samples, especially those containing ZE and TE, demonstrated a notable improvement in TPC levels, suggesting that the coatings contributed to the retention of bioactive compounds despite the thermal stress during frying. The TPC and antioxidant activity of potato strips before and after the frying process are presented in Figure 2a,b.

The lowest DPPH radical scavenging was observed in potato strips before the frying process (21.57%), and as a result of frying potatoes, their antioxidant activity increased significantly (*p* < 0.05). Although the phenolic compounds are oxidized and reduced during the frying process, during the thermal processes, the Maillard reaction occurs in foods, and the products resulting from this reaction indicate good antioxidant activity (Contreras‐Calderón et al. 2016). Coating potato strips with zedo gum solutions increased the antioxidant activity of fried samples by reducing the intensity of oxidation of active compounds compared to the control (*p* < 0.05). Since TE and ZE contain remarkable amounts of different bioactive compounds and have significant antioxidant activity, the use of coatings containing these essential oils led to a significant increase in the antioxidant activity of fried strips (*p* < 0.05). The DPPH radical scavenging values of fried strips coated with the solutions containing ZE and TE were 45.94% and 40.33%, respectively. In general, due to the higher primary TPC and antioxidant activity of ZE compared to TE, the strips coated with ZE showed higher TPC and antioxidant activity than those coated with TE. Carvacrol and thymol are the main phytochemicals of ZE, which have remarkable antioxidant activity (Ahmadi‐Dastgerdi et al. 2022). The increase in the antioxidant activity of fried potato chips containing rosemary essential oil compared to fresh potatoes has also been reported in the research of Trujillo‐Agudelo et al. (2020).

### 3.3 Oxidative Stability of Oils Extracted From Fried Potato Strips

#### 3.3.1 Peroxide Vale (PV)

Lipid oxidation is a critical factor influencing the quality, sensory attributes, and shelf life of fried foods. The primary oxidation products, hydroperoxides, form through the interaction of oxygen with unsaturated fatty acids, subsequently decomposing into secondary oxidation compounds, such as aldehydes and ketones, which are responsible for off‐flavors and rancidity (Chang et al. 2020). The extent of lipid oxidation is commonly assessed by measuring the peroxide value (PV), which reflects the initial stages of oxidative deterioration.

**FIGURE 3:**
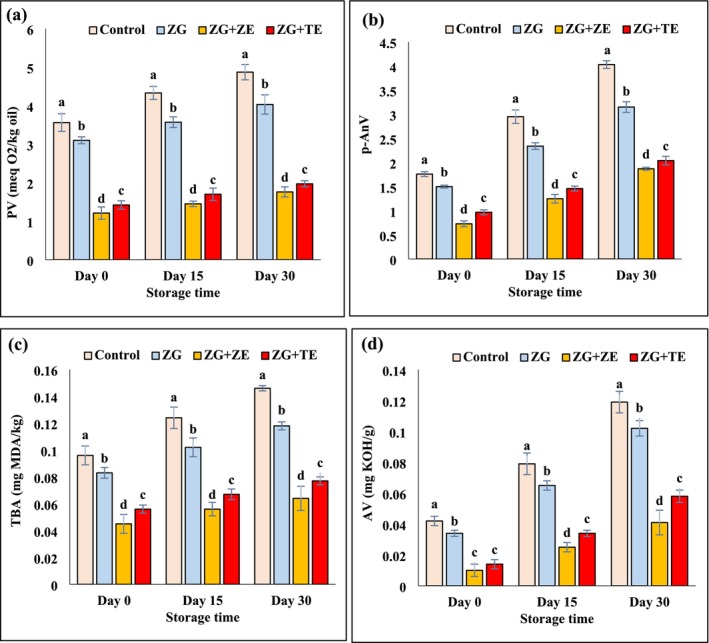
Changes in (a) POV, (b) *p*‐AnV, (c) TBA, and (d) AV values of fresh and fried potato strips during storage time. Different letters represent significant differences at the 5% level of probability among samples. ZG: Zedo gum‐based coating; ZG + ZE: Zedo gum‐based coating containing *Zataria multiflora* essential oil; ZG + TE: Zedo gum‐based coating containing tarragon essential oil. AV, acid value; *p*‐AnV, *para* anisidine value; POV, peroxide value; TBA, thiobarbituric acid.

In this study, the oil extracted from fried potato strips was analyzed over a 30‐day storage period to evaluate the impact of zedo gum‐based coatings containing *Z. multiflora* Boiss (ZE) and tarragon essential oil (TE) on lipid oxidation. As shown in Figure 3a, the initial PV of oils ranged from 1.42 to 3.56 meq/kg, indicating minimal oxidation at the start of storage. However, as time progressed, a significant increase in PV was observed across all samples (*p* < 0.05), with values reaching 1.76 to 4.87 meq/kg by the final day of storage. This increase reflects the gradual accumulation of hydroperoxides due to ongoing oxidative reactions, which are inevitable in fried products stored under ambient conditions.

Among all samples, the control (uncoated) potato strips exhibited the highest PV values throughout the storage period, confirming that uncoated fried foods are more susceptible to lipid oxidation. In contrast, potato strips coated with zedo gum‐based solutions demonstrated significantly lower PV values (*p* < 0.05), indicating that the coatings effectively delayed lipid oxidation. The most pronounced antioxidative effect was observed in strips coated with gum solutions containing ZE, followed by those containing TE. The superior performance of ZE can be attributed to its rich composition of phenolic compounds such as carvacrol and thymol, which are known to scavenge free radicals and inhibit oxidative chain reactions (Ahmadi‐Dastgerdi et al. 2022). These findings are consistent with previous studies demonstrating the protective role of plant‐derived antioxidants in lipid stabilization. Aly et al. (2021) reported that incorporating thyme powder into frying oil significantly reduced peroxide values (1.6–5.0 meq/kg) compared to unmodified control samples (13.8 meq/kg). Similarly, Manzoor et al. (2022) found that the addition of apple pomace enhanced the oxidative stability of soybean oil during French fry processing. Furthermore, Erol et al. (2022) observed that incorporating basil essential oil into frying oil mitigated oxidative degradation in fish fillets, aligning with our results. The efficacy of zedo gum‐based coatings in retarding lipid oxidation can be attributed to multiple mechanisms, including (1) Barrier Function: The edible coating acts as a physical barrier, reducing oxygen diffusion into the fried matrix and slowing down oxidation; (2) Phenolic Antioxidant Activity: The essential oils incorporated into the coatings provide additional radical‐scavenging capacity, limiting the formation of hydroperoxides and their subsequent breakdown into rancid compounds; and (3) Synergistic Effects: The combination of zedo gum and bioactive essential oils enhances the retention of endogenous antioxidants within the fried product, contributing to long‐term oxidative stability.

#### 3.3.2 Para Anisidine Value (p‐AnV)

The PV alone cannot represent the oxidation of oils, because this index shows the primary products of oxidation and does not represent the secondary products. Due to the decomposition of hydroperoxides at high temperature and during the storage period and formation of secondary products (such as aldehydes, alcohols, ketones, etc.), it is necessary to have tests such as para anisidine value (p‐AnV) and thiobarbituric acid (TBA) index, which are indicators of the progress of oxidation. P‐AnV is used to check the secondary products amounts resulting from oils and fats oxidation. Para‐anisidine reagent can react with carbonyl compounds resulting from oxidation (alpha‐ and beta‐alkenals) under acidic conditions (Abeyrathne et al. 2021). Factors such as oil type, temperature, and the presence of pro‐oxidants or antioxidants influence oxidation rates. Measuring both primary and secondary oxidation products provides a more comprehensive assessment of feed oil quality and potential health effects in animals (Winkler‐Moser et al. 2020).

The effect of active coating based on zedo gum on the p‐AnV of oils extracted from fried potato strips during a 30‐day storage period is shown in Figure 3b. In all the storage days, the control sample had the highest p‐AnV, and the strips coated with gum solutions had a lower p‐AnV compared to the control (*p* < 0.05). The addition of ZE and TE to the gum solution also led to an increase in the antioxidant activity of the coated solutions and significantly reduced the rate of lipid oxidation in fried potatoes and decreased the p‐AnV (*p* < 0.05). In all samples, with the passage of time due to the progress of the oxidation process and the breakdown of hydroperoxides and the production of secondary products of oxidation, the p‐AnV showed a significant increase (*p* < 0.05), such that the amount of this oxidation index was in the range of 0.73–1.76 on the first day of storage and reached 1.87–4.03 on the last day. In line with these results, Alizadeh et al. (2016) also reported that the use of rosemary extracts during the deep frying process of potato slices was able to significantly reduce the intensity of the increase in p‐AnV and PV of oils compared to the sample without extract (control). Jabri Karoui et al. (2016) stated that ZE contains various phenolic compounds such as cinnamic acid, rosmarinic acid, and carnosic acid and shows remarkable antioxidant activity. Further supporting these findings, a study by Manzoor et al. (2022) demonstrated that apple pomace extract and quercetin exhibited superior stability in mustard oil during deep frying compared to synthetic antioxidants like tertiary butylhydroquinone (TBHQ). These findings support that the bioactive compounds in the essential oils, when delivered in a gum matrix, interact with lipid substrates to limit oxidation reactions that would otherwise lead to elevated anisidine values in fried products (Aghababaei et al. 2024).

#### 3.3.3 TBA Index

TBA is another major oxidation index in oils and fats, which is used to measure secondary products of lipid oxidation such as malondialdehyde (MDA). In Figure 3c, the changes in the TBA values of oils extracted from fried potato strips during the storage period are given. At the beginning of the storage period, the highest amount of TBA was obtained in the control sample (0.096 mg MDA/kg) and the sample coated with zedo gum solution without essential oils was next (0.083 mg MDA/kg). The lowest amounts of this oxidation index were observed in the samples coated with gum solutions containing ZE (0.045 mg MDA/kg) and TE (0.056 mg MDA/kg). During the storage period, the TBA values of all samples increased, but the highest rate of increase of this oxidation index was for the uncoated sample (*p* < 0.05). On the last day of storage, the control sample (0.146 mg MDA/kg) and the sample coated with gum solution containing ZE (0.064 mg MDA/kg) had the highest and lowest TBA values, respectively. Overall, coating potato strips with zedo gum solutions before the frying process increased the oxidation stability of the oils in the fried strips by reducing the availability of oxygen, increased the oxidative stability of oils, and significantly reduced the values of oxidation indexes in the treated samples compared to the uncoated sample (*p* < 0.05). Since ZE and TE contain significant amounts of bioactive and phenolic compounds, they show high antioxidant activity and improve the oxidative stability of fried potatoes. Antioxidants generally delay oxidation reactions by donating hydrogen atoms to the free radicals. In fact, polyphenols are able to scavenge free radicals, especially proxy radicals, which are the most key intermediate chain reactants, and cause the termination of the oxidation reaction cycle and decrease the rate of formation of primary and secondary products resulting from lipid oxidation (Peasura et al. 2015). The improvement of the oxidative stability of fried food products during the storage period due to the addition of ZE has also been confirmed by other researchers (Ahmadi‐Dastgerdi et al. 2022; Tokur et al. 2021). The noticeable effect of edible coatings containing TE on increasing the oxidative stability of food products has also been reported by previous researchers (Farsanipour et al. 2020; Zhang et al. 2020). In agreement with the results of the present research, in the study of Wang, Chen, et al. (2020), Wang, Meng, et al. (2020), *Mentha spicata cv. Henanshixiang* essential oil was able to reduce the TBA values of oil during the Chinese *Maye* frying. Also, Jouki and Khazaei (2022), investigating the effects of active paste coatings enriched with betel nut gum and carvacrol microcapsules on oil absorption and quality loss in nuggets during frying, found that the primary and secondary oxidation products in the fried nugget samples showed that the thiobarbituric acid (TBA) levels in the control samples were 1.35 mg MDA/kg. The results of this study indicated that the highest reduction in TBA was 37.04% for the QSG‐coated samples containing 1% carvacrol microcapsules.

### 3.4 Acid Value (AV)

The AV is an indicator that shows the amount of free fatty acids in edible fats and oils. Free fatty acids are produced by the hydrolysis of triglycerides, and a small amount of them is also obtained as a result of the oxidation reaction (Wang, Meng, et al. 2020). The results of the AV of oils (Figure 3d) showed that at the beginning of the storage period, the control sample had the highest AV, and by coating the potato strips with zedo gum solutions, a reduction in the hydrolysis of triglycerides occurred during the frying process and therefore the AV decreased significantly compared to the control (*p* < 0.05). Preventing the exit of moisture during the frying process through the use of hydrocolloid coatings is the reason for reducing the intensity of triglycerides hydrolysis and reducing the AV of fried foods. The presence of antioxidant compounds of TE and ZE in the coating solutions also improved the stability of the oil in fried potatoes and reduced the AV significantly (*p* < 0.05). The AV of the oils on the first day of storage was in the range of 0.010–0.042 mg KOH/g, and with the passage of time due to the hydrolysis of triglycerides, the AV of the oils gradually increased (*p* < 0.05) and reached their highest values on the last day (0.064–0.146 mg KOH/g). Similarly, Boroujeni and Hojjatoleslamy (2018) also reported that the fried potato chips in oils containing *Myrtus communis* and *Thymus carmanicus* essential oils had a lower AV compared to the control sample, and an increase in the AV was observed in all samples during the storage period.

Abdollahi et al. (2023) studied the oxidation results of fried zucchini treated with apricot gum over 30 days. The initial acid values ranged from 0.08 to 0.09 mg KOH/g oil, indicating high quality. However, the AV increased significantly during storage, with the control sample having a higher value. The highest and lowest AV were observed in the control and 12% gum samples, respectively. The AV did not exceed the standard limit during storage (1–4 mg KOH/g oil). In contrast to Abdollahi's study, our study showed a similar trend of AV increase over time for fried potato strips. However, the zedo gum‐coated samples showed a significant reduction in AV compared to the control group, suggesting the effectiveness of zedo gum coatings in mitigating hydrolysis and oxidation processes during frying and storage. The reduction in acid value was more pronounced due to the presence of essential oils (TE and ZE), which reduced free fatty acid production and enhanced oil stability, further proving the efficacy of bioactive coatings in preserving oil quality.

### 3.5 Acrylamide Content

**FIGURE 4:**
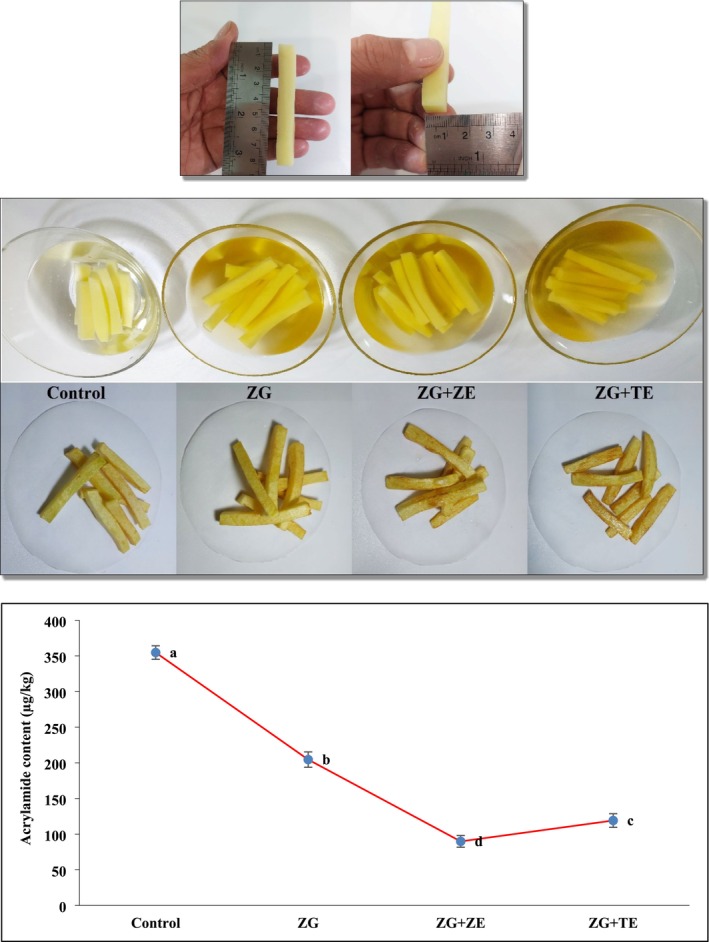
Preparation steps, appearance of final products, and acrylamide content of fried potato strips. Different letters represent significant differences at the 5% level of probability among samples. ZG: Zedo gum‐based coating; ZG + ZE: Zedo gum‐based coating containing *Zataria multiflora* essential oil; ZG + TE: Zedo gum‐based coating containing tarragon essential oil.

The acrylamide content of fried potato strips is shown in Figure 4. The uncoated sample (control) had the highest acrylamide content (354.73 μg/kg), and by coating the potato strips with the zedo gum solution before the frying process, a significant decrease in the acrylamide content of the strips was observed (*p* < 0.05). The lowest amount of acrylamide was for the strips coated with the solution containing ZE (89.84 μg/kg) and the strips coated with the solution containing TE were next (199.10 μg/kg). The selection of ZE and TE essential oils was based on their strong antioxidant potential, which is crucial in reducing acrylamide formation. These essential oils are rich in bioactive compounds such as thymol, carvacrol, estragole, and anethole, which have been reported to inhibit Maillard and lipid oxidation reactions, thereby mitigating acrylamide production. Additionally, their antimicrobial and sensory‐enhancing properties make them particularly suitable for application in food coatings. Previous studies have explored various natural antioxidants for acrylamide reduction, but limited research has investigated the synergistic effect of ZE and TE essential oils in a zedo gum‐based coating for fried potato products, highlighting the novelty of this study.

Trujillo‐Agudelo et al. (2020) conducted a factorial experimental design to evaluate the effect of an edible coating made from whey protein and rosemary extract on the reduction of acrylamide in potato chips. Their results showed that acrylamide content varied between 130 and 650 μg/kg, which was significantly affected by frying temperature (*p* < 0.05). Overall, natural antioxidants are able to reduce the intensity of acrylamide formation in fried products due to the presence of different bioactive compounds, especially phenolic compounds. Although acrylamide precursors such as reducing sugars (e.g., glucose and fructose) and free asparagine were not directly measured in this study, previous research has shown that natural antioxidants, particularly phenolic compounds, can modulate acrylamide formation by interacting with these precursors in Maillard and lipid oxidation reactions. One of the mechanisms proposed to reduce acrylamide by phenolic compounds is the inhibition and trapping of Deoxy‐2‐3 hexulose sugars, which are of interest as an intermediate compound in Maillard reaction (Tesby et al. 2018). The reduction of acrylamide formation by natural antioxidants in general is probably due to the fact that these active compounds can interact with the precursors of acrylamide formation in the two important reactions of Maillard and lipid oxidation, which leads to the formation of acrylamide. In the Maillard reaction, the reducing sugar part reacts with the conjugated systems of polyphenols with antioxidant activity, and in this way, it prevents the reaction of asparagine with sugar (Morales et al. 2014). In the lipid oxidation reaction, during the destruction of these macromolecules, acrolein is formed, which turns into acrylic acid or acrylic radical during the oxidation reaction (Becalski et al. 2003). These intermediates can subsequently react with nitrogen‐containing compounds to form acrylamide. Antioxidants in ZE and TE coatings may contribute to acrylamide reduction by scavenging free radicals and inhibiting lipid oxidation, thereby minimizing the availability of acrylamide precursors. Both of these intermediates eventually form acrylamide through reaction with nitrogen sources. By scavenging free radicals and inhibiting acrolein oxidation, ZE and TE essential oils play a pivotal role in reducing acrylamide formation in fried potato strips. Antioxidants can block the acrolein oxidation and thereby reduce the formation of acrylamide (Morales et al. 2014).

In line with the results of the present research, Abu‐Almaaly and Al‐Musawi (2021) also reported that the acrylamide content of potato chips was 0.522 mg/kg and the immersion of potatoes in thyme extract led to a significant decrease in the acrylamide content of the samples, so that at 1% concentration of thyme extract, the amount of acrylamide in the chips was decreased to 0.236 mg/kg. Mekawi et al. (2019) also found that the immersion of potato chips in pomegranate extract nanoparticles led to a significant decrease in the acrylamide content of the treated samples, so that the control sample contained 1.67 mg/kg acrylamide and its amount reached 0.019 mg/kg in the sample treated with extract. In another study, coating of potato chips with hydrocolloid coatings based on Arabic gum and soluble soybean polysaccharides reduced the acrylamide content in chips and improved the quality of the products (Torabi et al. 2017).

### 3.6 Hardness of Fried Potatoes

The hardness of the texture is one of the important parameters related to the quality of fried potato strips that have a noticeable effect on the acceptance of the final products by consumers. Various studies have shown that different essential oil coating formulations can either increase or decrease the hardness of French fries, with the effects varying depending on the type of coating and processing conditions. For example, composite formulations containing zein, alginate, and potato starch produced reasonable firmness under low‐humidity conditions (Emragi et al. 2022), while a chitosan‐based coating with cinnamon oil decreased measured firmness by 3.80 N, with 0.6% cinnamon oil leading to a decrease of 10.20 N during cold storage (Sarengaowa et al. 2022). In contrast, a whey protein‐based coating with rosemary extract (whey protein: 5%–11%, rosemary extract: 0%–2%) improved the firmness (ranged between 0.5 and 5.7 N) of fried potato chips under frying conditions (170°C–190°C) (Trujillo‐Agudelo et al. 2020).

**FIGURE 5:**
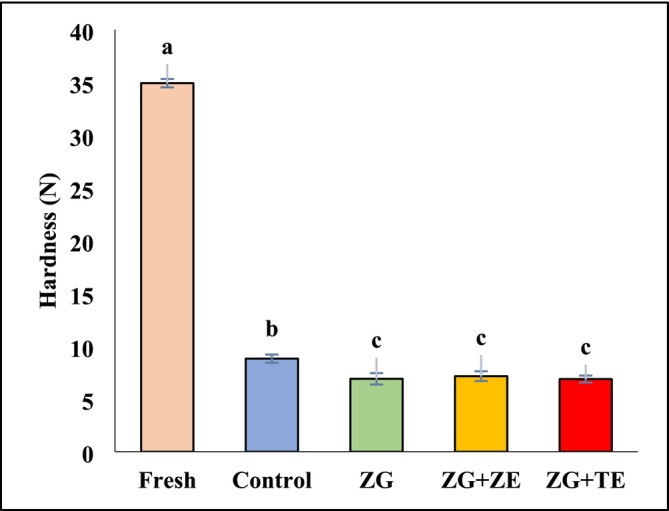
Hardness values of fresh and fried potato strips. Different letters represent a significant difference at the 5% level of probability among samples. ZG: Zedo gum‐based coating; ZG + ZE: Zedo gum‐based coating containing *Zataria multiflora* essential oil; ZG + TE: Zedo gum‐based coating containing tarragon essential oil.

The effect of the zedo gum‐based edible coatings on the hardness of the fresh and fried potato strips is shown in Figure 5. The fresh potato strips exhibited the highest hardness (34.93 N) due to their intact cellular structure and high moisture content. However, the frying process significantly reduced the moisture content and created a brittle crust on the surface, leading to a decrease in internal resistance and an overall reduction in hardness (*p* < 0.05). The coating process with zedo gum‐based solutions also had a significant effect on the hardness of the fried strips (*p* < 0.05). The highest hardness was recorded for the control sample (8.82 N), whereas the application of edible coatings significantly reduced the hardness of the fried samples compared to the control (*p* < 0.05). The coated fried strips exhibited hardness values in the range of 6.89–7.16 N, with no significant difference among the different coated samples. Since the use of hydrocolloid coatings has resulted in better moisture in fried strips compared to the control, their lower hardness compared to the uncoated sample is not far from expected. Similarly, Noshad et al. (2017) reported that fried shrimp coated with quince seed mucilage‐based coatings containing green tea extract exhibited lower hardness than uncoated samples due to their higher moisture content. Boroujeni and Hojjatoleslamy (2018) also observed a slight reduction in the hardness of fried potato strips containing *Thymus carmanicus* compared to the control.

### 3.7 Color of Fried Potatoes

Color is one of the important quality characteristics of food products that is evaluated by consumers. During the frying process, the simultaneous transfer of heat and mass causes physical and chemical changes in the fried foods, and in this case, the color of the product is also affected (Falguera et al. 2011). The results of examining the color indexes of fresh and fried potato strips are given in Table 1. The frying process had a significant effect on the color of potato strips (*p* < 0.05), so that the highest L* (84.35) and the lowest a* (−0.36, indicating a very slight shift toward the green spectrum but not visually perceived as green) and b* (3.65) were observed in fresh strips. Among all C* values, a significant difference was observed at the 5% level (*p* < 0.05), indicating that coating, coating type, and heat treatment have a significant impact. Regarding the h° value, a significant difference was only observed when heat was applied; therefore, no significant difference was observed among the coated samples (*p* > 0.05). However, there was a significant difference between the control sample and all fried samples (*p* < 0.05). The presence or absence of coating (e.g., in the control sample) indicates that coating is not a factor in changing the h° value, and heat application through frying has a significant effect on potato slices during the process.

**TABLE 1:** Color indexes of fresh and fried potato strips.

Samples	L*	a*	b*	C*	*h*°
Fresh	84.35 ± 0.76^a^	−0.36 ± 0.08^b^	3.65 ± 0.15^b^	6.72 ± 0.15^e^	−1.47 ± 0.11^b^
Control	68.72 ± 0.95^b^	0.54 ± 0.03^a^	15.54 ± 0.07^a^	120.89 ± 1.07^d^	1.53 ± 0.15^a^
ZG	65.11 ± 0.89^c^	0.61 ± 0.05^a^	16.65 ± 0.13^a^	138.79 ± 1.01^c^	1.53 ± 0.17^a^
ZG + ZE	65.06 ± 1.11^c^	0.57 ± 0.02^a^	16.83 ± 0.11^a^	141.78 ± 0.97^b^	1.53 ± 0.09^a^
ZG + TE	64.90 ± 0.55^c^	0.60 ± 0.06^a^	17.07 ± 0.17^a^	145.87 ± 0.73^a^	1.53 ± 0.06^a^

*Note:* Values represent mean (*n* = 3) ± SD. Different letters represent significant difference at 5% level of probability among samples. ZG: Zedo gum‐based coating; ZG + ZE: Zedo gum‐based coating containing *Zataria multiflora* essential oil; ZG + TE: Zedo gum‐based coating containing tarragon essential oil. L*, a*, b*, Chroma (C*), and Hue angle (*h*°) are color parameters.

Coating of potato strips with zedo gum solutions also showed a significant effect on the brightness and yellowness of fried strips (*p* < 0.05); however, it did not show any effect on the redness of strips. The values of L*, a*, and b* indices of fried potato strips were in the range of 64.90–68.72, 0.54–0.61, and 15.54–17.07, respectively. The brightness of coated strips was lower than that of uncoated strips (control), but compared to the control, they had a higher yellowness. A controlled laboratory study tested coatings made from carboxymethyl cellulose (CMC) or gum arabic combined with olive or nettle leaf extracts and measured their effect on the color indices of fried potatoes. Under frying conditions of 180°C ± 2°C for 10 min, the coatings decreased the L* value, indicating reduced brightness, and increased the a* value, indicating greater redness. No information was provided on changes to the b* value (yellowness). Both CMC‐ and gum arabic‐based formulations resulted in similar color shifts (Mia Kurek et al. 2022). The decrease in the brightness of the strips coated with zedo gum solutions is due to the increase in the intensity of the Maillard reactions of the samples due to the carbohydrate nature of zedo gum. The Maillard reaction, with the production of brown pigments, reduces the color brightness of fried products and improves the intensity of yellowness (Eslampour and Hosseini 2018). Soroushfard et al. (2021) also reported that immersing potato slices in turmeric extract improved the color of fried potatoes by reducing the intensity of the non‐enzymatic reaction of Maillard. Salehi et al. (2021) showed that fried and coated potato strips with seed mucilage and xanthan gum had higher redness and yellowness and lower brightness than the control strips.

### 3.8 Sensory Evaluation of Fried Potatoes

The sensory properties of fried potato strips, including color, flavor, texture, and overall acceptability, were evaluated using a 5‐point Hedonic test, and the results are presented in Table 2. In terms of color, flavor, and overall acceptability, potato strips coated with the zedo gum solution containing tarragon essential oil (TE) received the lowest score. However, in terms of texture, no statistically significant difference was observed between the different samples. Despite the lower sensory scores of potato strips containing TE, all the samples studied in this research were acceptable in terms of various sensory characteristics, receiving good scores and demonstrating good sensory acceptance. These findings are consistent with the results of Mia Kurek et al. (2022), where polymer and extract type did not significantly affect the firmness and stickiness of the coated samples before frying. Their study also indicated that increasing the amount of extract in coatings led to higher crispness and lower oiliness, which was considered a positive effect compared to the control.

**TABLE 2:** Sensory characteristics of fried potato strips.

Samples	Color	Flavor	Texture	Overall acceptability
Control	4.30 ± 0.24^ab^	4.50 ± 0.26^a^	4.30 ± 0.24^a^	4.40 ± 0.25^a^
ZG	4.70 ± 0.24^a^	4.50 ± 0.26^a^	4.60 ± 0.25^a^	4.50 ± 0.26^a^
ZG + ZE	4.50 ± 0.26^ab^	4.90 ± 0.16^a^	4.50 ± 0.26^a^	4.80 ± 0.21^a^
ZG + TE	4.20 ± 0.21^b^	3.80 ± 0.21^b^	4.50 ± 0.26^a^	4.00 ± 0.00^b^

*Note:* Values represent mean (*n* = 3) ± SD. Different letters represent significant difference at 5% level of probability among samples. ZG: Zedo gum‐based coating; ZG + ZE: Zedo gum‐based coating containing *Zataria multiflora* essential oil; ZG + TE: Zedo gum‐based coating containing tarragon essential oil.

Similarly, in our study, Abu‐Almaaly and Al‐Musawi (2021) found that immersion of potato chips in thyme extracts did not negatively affect the sensory characteristics of the chips, and the samples treated with this extract exhibited higher sensory acceptance than the control. Furthermore, Aly et al. (2021) observed that fried potatoes in sunflower oil containing thyme received high sensory scores and were deemed acceptable. In our study, while the zedo gum coatings with essential oils significantly reduced the acrylamide content and oil uptake, the sensory evaluation revealed no major negative impacts on texture, aligning with the findings of Mia Kurek et al. (2022), where higher extract amounts enhanced crispiness and reduced oiliness, with no significant difference in texture.

Moreover, a very strong correlation was found between browning intensity and crispiness (*r* ^2^ = 0.90) in the study of Mia Kurek et al. (2022), which aligns with our results indicating that the coatings did not negatively affect the appearance or texture of the fried potato strips. Our study also corroborates the findings by Zokaei et al. (2020), who observed improved sensory characteristics in potato chips coated with caseinate and pectin solutions containing *Z. multiflora* extracts. Thus, despite the slight differences in the sensory characteristics of coated potato strips, all the samples demonstrated good sensory acceptability, highlighting the effectiveness of zedo gum‐based coatings in preserving the quality and sensory attributes of fried products.

## 4 Conclusion

In this research, the effect of active coatings based on zedo gum containing ZE and TE on the quality of fried potato strips was studied, and the results showed that by coating potato strips with active coatings containing essential oils, the antioxidant activity of fried strips improved and the oxidative stability of the samples increased significantly. Overall, the antioxidant activity of ZE was higher than TE. Furthermore, coating with zedo gum solutions also reduced the rate of moisture loss, oil uptake, and acrylamide formation in fried potato strips. According to the results obtained in this research, it can be concluded that due to the improvement of the quality, oxidative stability, and sensory acceptance of fried and coated potato strips with zedo gum solutions containing ZE and TE, it is possible to use these edible and active coatings for maintaining the quality of potato strips during the deep‐fat frying process, and the best treatments in this research are the coated potato strips with gum solution containing ZE.

## Author Contributions

**Niyoosha Khajeh:** formal analysis (equal), investigation (equal), methodology (equal), writing – original draft (equal). **Hamid Babapour:** data curation (equal), formal analysis (equal), investigation (equal), software (equal), writing – original draft (equal). **Bahram Hassani:** data curation (equal), investigation (equal), validation (equal). **Abdorreza Mohammadi Nafchi:** data curation (equal), investigation (equal), validation (equal). **Leila Nouri:** conceptualization (equal), data curation (equal), investigation (equal), supervision (equal). **Ahmadreza Abedinia:** data curation (equal), software (equal), supervision (equal), writing – review and editing (equal).

## Ethics Statement

The authors have nothing to report.

## Consent

The authors have nothing to report.

## Conflicts of Interest

The authors declare no conflicts of interest.

## Data Availability

Available data will be expressed on request.
